# Strategies for the introduction of human papillomavirus vaccination: modelling the optimum age- and sex-specific pattern of vaccination in Finland

**DOI:** 10.1038/sj.bjc.6603575

**Published:** 2007-01-23

**Authors:** K M French, R V Barnabas, M Lehtinen, O Kontula, E Pukkala, J Dillner, G P Garnett

**Affiliations:** 1 Department of Infectious Disease Epidemiology, Imperial College, Norfolk Place, Paddington, London, W2 1PG, UK; 2 HIV Vaccine Trials Network, Seattle, USA; 3 National Public Health Institute, Finland; 4 Family Federation of Finland, Finland; 5 Finnish Cancer Registry, Institute for Statistical and Epidemiological Cancer Research, Finland; 6 Lund University, Sweden

**Keywords:** HPV, vaccination, modelling

## Abstract

Phase III trials have demonstrated the efficacy of human papillomavirus (HPV) vaccines in preventing transient and persistent high-risk (hr) HPV infection and precancerous lesions. A mathematical model of HPV type 16 infection and progression to cervical cancer, parameterised to represent the infection in Finland, was used to explore the optimal age at vaccination and pattern of vaccine introduction. In the long term, the annual proportion of cervical cancer cases prevented is much higher when early adolescents are targeted. Vaccinating against hr HPV generates greater long-term benefits if vaccine is delivered before the age at first sexual intercourse. However, vaccinating 12 year olds delays the predicted decrease in cervical cancer, compared to vaccinating older adolescents or young adults. Vaccinating males as well as females has more impact on the proportion of cases prevented when vaccinating at younger ages. Implementing catch-up vaccination at the start of a vaccination programme would increase the speed with which a decrease in HPV and cervical cancer incidence is observed.

Human papillomavirus (HPV) is a common sexually transmitted agent (Trottier and Franco, 2006) and persistent infection with high risk (hr) types of HPV, most notably types 16 and 18, is the most important risk factor for cervical cancer (Ho *et al*, 1998). Rates of invasive cervical cancer (ICC) in fertile aged women in Finland declined significantly from 1960 to 1990 (Figure 1), mostly as a result of the national screening programme. However, cervical cancer incidence in Finnish women aged 30–39 years is now four times higher than 15 years ago (2.9/100 000 in 1986–1990; 9.8/100 000 in 2000–2004: Finnish Cancer Registry, 2006). This is probably due to an increase in incidence and prevalence of hr HPV types in young (23–28 year old) Finnish women (Laukkanen *et al*, 2003; Lehtinen *et al*, 2006).

In phase III trials, two HPV virus-like particle vaccines have been shown effective in preventing incident and persistent HPV16 and 18 infection and associated precancerous lesions, with reported efficacies in the region of 90–100% (Koutsky *et al*, 2002; Harper *et al*, 2004, 2006; Villa *et al*, 2005; Mao *et al*, 2006). The vaccines could prevent around 70% of all cervical cancer (Munoz *et al*, 2003). One vaccine has recently been licensed for use in the US and in Europe. However, there remain important questions about how a HPV vaccine should be used at a population level (Lowndes and Gill, 2005). These include: the age chosen for vaccination, whether the vaccine is given to female subjects only or to female and male subjects and whether a catch-up vaccination campaign should accompany the introduction of routine vaccination. As mathematical models provide a framework for exploring these questions (Garnett, 2002), we have examined these questions with a model of single-type HPV using the observed epidemiology of HPV in Finland (Barnabas *et al*, 2006).

## MATERIALS AND METHODS

In earlier work, we used sexual behaviour data along with HPV seroprevalence trends in pregnant women to parameterise a mathematical model of HPV16 occurrence in Finland (Barnabas *et al*, 2006). This provided a framework which we developed further to explore the impact of age at first sexual intercourse, and the age of vaccination on occurrence of HPV16 and associated cervical cancer cases. The model stratified the population by 5-year age groups. To explore aspects of age of vaccination, we adapted the model to represent single years of age, with a gradual increase in the proportion of adolescents who were sexually active. The earlier detailed description of the model is still applicable and is summarised here. The population was stratified into age, sex and sexual activity classes with defined rates of sexual partner change (Barnabas *et al*, 2006). The model describes the transmission of virus between the sexes and the flow of incident cases in women from the acquisition of asymptomatic HPV infection, through premalignant disease to ICC, with most HPV infections regressing spontaneously. It was also assumed that regression leading to the clearance of infection results in lifelong acquired immunity. The rates of regression and progression between stages of disease used in the earlier model (Barnabas *et al*, 2006) were adapted to allow linear increase and decrease in rates with age rather than a sudden increase in progression rates at age 35. Cervical screening was based on the reported age-specific proportion of women screened through the national Finnish screening programme as in the earlier model (Barnabas *et al*, 2006). Screening starts at the age of 25 or 30 years (in the model at age 25) and continues up to age 60 with a screening interval of 5 years. Successful identification of HPV infection and associated precancerous lesions and its treatment was assumed to result in lifelong acquired immunity. It was assumed that screening did not change after the introduction of vaccination. A simple representation of infection in men was used, assuming they could be susceptible, infected or immune.

The proportion of the population sexually active at each age was set according to data from the Family Federation of Finland, with the percentages sexually active by age shown in Table 1. Although data on the age of sexual debut from 12 years and older were available, partner change rates were not available for 12–14-years-old and we assumed that for this age group partner change rates were similar to that at age 15–19 years.

We explored the impact of vaccination on cases of cervical cancer. For different ages at vaccine delivery (12, 15, 18 and 21), we compared the predicted proportion of cervical cancer caused by HPV16 prevented by vaccination of 70% of female subjects introduced in 2008. For simplicity, we assumed that the vaccine has 100% efficacy with lifelong duration of protection and that the vaccine had no effect on those already infected. The additional proportion of cervical cancer cases prevented by vaccinating male as well as female subjects at different coverages, and by different strategies of catch-up vaccination at the start of a campaign was also examined. We assumed 3 years of catch-up vaccination at the start of a vaccination campaign aimed at 12-years-old, corresponds to vaccinating at ages 12, 13, 14 and 15 in the first year of the campaign. We explored four catch-up vaccination strategies corresponding to 3, 6, 9 and 12 additional years age cohorts vaccinated in the first year.

## RESULTS

**Figure 2:**
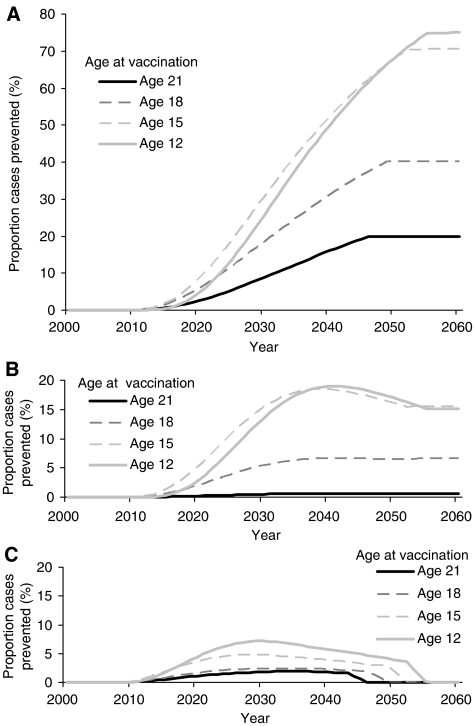
Proportion of annual incident HPV16-associated cervical cancer cases prevented with different ages at vaccination if coverage is 70% of females only (**A**). Additional proportion of total HPV16-associated cervical cancer cases prevented for each age at vaccination by vaccinating male as well as female subjects with the same 70% coverage (**B**) and catch-up vaccination that involves vaccinating three additional ages in the first year of vaccination with the same 70% coverage (**C**).

The model predicts that vaccinating young adolescents at 12 years of age, compared with 15-years-old, delays the impact of immunisation on HPV16-associated cervical cancer incidence (Figure 2A). Before age 16, only a small proportion of the population is sexually active and transmitting the infection (Table 1); it is as the vaccinated cohort ages and becomes sexually active that an impact on incidence is observed. Infection before vaccination results in the impact of vaccination declining as the age of vaccination increases when more of the population is sexually active and HPV incidence is higher. The predicted peak HPV incidence in women (and in men), generating the observed pattern of disease before vaccination, is seen at age 20. Once the full impact of vaccination is reached, the annual proportion of cases of HPV16-associated cervical cancer prevented is 20% if vaccination occurs at age 21; 40% if this occurs at age 18; 67% if it occurs at age 15; and 68% if it occurs at age 12. There is relatively little difference, especially in the long term between vaccinating at 12 and 15 years of age (Figure 2A).

The predicted impact of vaccinating male subjects in addition to female subjects depends upon the age of vaccination and the coverage. At younger ages, the number of additional cases prevented is greater (Figure 2B). In the long-term, vaccinating male as well as female subjects at ages 12 or 15 annually prevents an additional 15.1 and 15.5% of cases, respectively. If vaccination occurs at age 21, vaccinating male subjects has very little effect on incidence of cervical cancer, in the long-term preventing annually an additional 1% of cases. The benefit of vaccinating both sexes, in terms of the proportion of cervical cancer cases prevented, increases with vaccination coverage, peaking at 50% coverage (Table 2). If vaccination occurs at age 12, vaccinating male as well as female subjects at 30 or 70% coverage prevents an additional 15% of cases, whereas at 50% coverage an additional 18% of cases are prevented annually.

Three years of catch-up vaccination at the start of a campaign also has more impact at younger ages of vaccination (Figure 2C) because the ages included in the catch-up occur before the age of peak incidence. In the first 10 years after the start of the vaccination campaign, 3 years of catch-up prevents an additional 6% of cervical cancer cases if vaccination occurs at age 21, 10% if this occurs at age 18, 18% if it occurs at age 15 and 15% if it occurs at age 12.

Although the relative impact of 3-year catch-up vaccination on the proportion of cases prevented annually in the long term is highest if vaccination occurs at age 12 (Figure 2A and C), the absolute *cumulative* number of cases of HPV16-associated cervical cancer prevented by 2055 both with and without the 3-year catch-up is highest if vaccination occurs at age 15 (Table 3). In the former, the impact of vaccination takes 3 years longer to be realised as only a small proportion of the population is sexually active before age 15. In contrast, when vaccinating at ages 18 or 21, because a large proportion of infections occur before vaccination, the number of cumulative cases prevented is lower.

**Figure 3:**
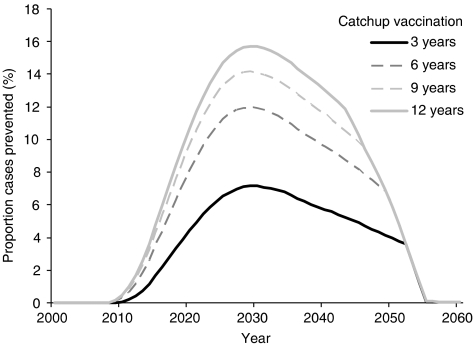
Additional proportion of total HPV16-associated cervical cancer cases prevented by vaccination with different numbers of ages included in a catch-up programme. Vaccination is at age 12 with 70% coverage of female subjects only and catch-up applied in the first year.

Increasing the range of ages included in a catch-up vaccination programme aimed at 12-years-old beyond 6 years, yields diminishing returns (Figure 3). A 6-year catch-up programme, vaccinating ages 12–18 inclusive, prevents an additional 11% of HPV16-associated cervical cancer cases in the first 10 years compared with a 3-year catch-up programme, vaccinating ages 12–15 inclusive. A further 3 years, vaccinating ages 12–21, prevents an additional 5% and another 3 years, vaccinating ages 12–24, prevents an additional 3% of cases in the first 10 years.

**Figure 4:**
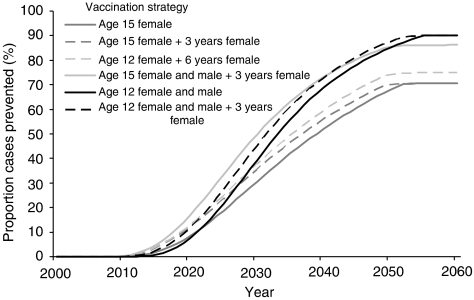
The impact of five possible vaccination strategies on the proportion of HPV16- associated cervical cancer cases prevented. Age 15 Female is a programme aimed at vaccinating 15-year-old female subjects annually, Age 15 Female+3 years Female is a programme vaccinating 15–18-year-old female subjects in the first year and 15-year-old female subjects in subsequent years, Age 12 Female+6 years Female is a programme vaccinating 12–18-year-old female subjects in the first year and 12- year-old female subjects in subsequent years, Age 15 Female and Male+3 years Female is a programme vaccinating 15–18-year-old female subjects and 15-year-old male subjects in the first year and 15-year-old male and female subjects in subsequent years, Age 12 Female and Male is a programme vaccinating 12-year-old female and male subjects annually and Age 12 Female and Male+3 years Female is a programme vaccinating 12–15-year-old female and 12 male subjects in the first year and 12-year-old male and female subjects in subsequent years.

Figure 4 shows the impact of selected possible vaccination strategies on HPV16-associated cervical cancer cases. With a vaccination programme aimed at 15-year-old female subjects, a predicted 71% cases of HPV16-associated cervical cancer could be prevented annually by 2055, when the full impact of the campaign is realised (Figure 4). If a 3-year catch-up vaccination is included (so the first year of the programme involves vaccinating 15–18-year-old female subjects), the proportion of cases prevented in the long term is not changed, but the impact is observed sooner (Figure 4). If the age at vaccinating females is lowered to age 12 and 6 years of catch-up vaccination is included, 75% of cases could be prevented each year by 2055 (Figure 4). If vaccination is aimed at 15-year-old male and female subjects, with a 3-year catch-up vaccination in females only, a predicted 86% of HPV16-associated cervical cancer cases could be prevented annually by 2055 (Figure 4). If, alternatively, vaccination is aimed at 12-year-old males and females, with or without catch-up vaccination, the annual proportion of cases prevented by 2055 is 90%, though without catch-up the impact takes longer to be realised (Figure 4).

## DISCUSSION

Modelling the dynamics of HPV infection and impact of vaccination based on empirical data (Koutsky *et al*, 2002; Harper *et al*, 2004; Villa *et al*, 2005; Barnabas *et al*, 2006; Harper *et al*, 2006) allows us to predict the patterns of infection and disease associated with a range of strategies. Qualitative rules emerge: (1) vaccinating before sexual debut maximises the long-term impact of vaccination; (2) catch-up programmes can speed impact and decrease the cumulative number of cases; (3) vaccinating male subjects prevents additional cases, but the proportion is smaller than the concomitant increase in the fraction vaccinated. These results and their innovative combination can help in determining policy, but should be considered in the relevant context, both in terms of patterns of risk behaviour and opportunities for vaccination. What coverage is possible in the different age groups and who will deliver the vaccine and at what cost?

Our model generates numerical results and it is tempting to regard the predictions as definite. However, there is great uncertainty concerning both model structure and parameter values. We have, for simplicity, only considered one HPV type (HPV16), which is present in at least half of the cervical cancers. There may be differences in the oncogenic potential of the different hrHPV types, but with appropriate parameters the same model could be used to explore other types. More significantly, we have assumed complete naturally acquired immunity on clearance of infection. The age of incidence of HPV infection and cervical neoplasia suggest that a naturally derived type-specific immunity occurs, and observational studies suggest that there is also cross reactive natural immunity (Luostarinen *et al*, 1999, 2004), but their impact and duration are uncertain. If we assumed no natural immunity or a short duration of natural immunity, we would expect the impact of vaccination to be greater and additional coverage to add less. We would also expect to see higher rates of incidence of HPV in older women, possibly increasing the value of vaccinating at older ages. In contrast, we used reported sexual behaviours (Haavio-Mannila *et al*, 2001) to describe the distribution of sexual activity, which limited the extremes of behaviour of the model population. A greater variance in risk would reduce the impact of a given vaccination coverage and make additional target groups more attractive.

We assume the vaccine is 100% effective with lifelong duration of protection. A lower vaccine efficacy would result in reduced proportions of cases prevented but the difference between vaccination strategies would not be affected. However, if duration of protection is lower the proportion of cases prevented by different strategies may be altered, particularly if the duration of protection means that the vaccinated cohort would cease to be protected by the time they reach the age of peak incidence. In addition, the vaccine was assumed to have no effect on individuals already infected with HPV. Although it has not yet been shown conclusively that vaccines have any therapeutic effect, if they significantly reduce infectiousness in those already infected, vaccination at older ages would prevent more cases than we have observed as a result of reductions in transmission. This is an important consideration that needs further research.

So far the cost-effectiveness of HPV vaccination has not really been considered in relation to implementing HPV into the national vaccination programme (Goldie *et al*, 2004; Taira *et al*, 2004). The logical extension of this analysis would be to attach costs to both disease and vaccination. This would allow a cost-effectiveness analysis including the incremental benefits associated with the incremental costs of extending vaccination. Cost-effectiveness analyses usually discount future health benefits at a rate per year, which may nullify the observed advantage of vaccination at age 12 over age 15. The advantage of using a transmission-dynamic model like ours in a cost-effectiveness analysis is that it allows the investigation of the relative impact upon incidence of vaccinating different groups, especially with respect to vaccinating females *vs* both sexes. The current analysis allows qualitative insights into how to focus vaccination campaigns, but more detailed work is required to determine the feasibility and costs of such campaigns.

## Figures and Tables

**Figure 1:**
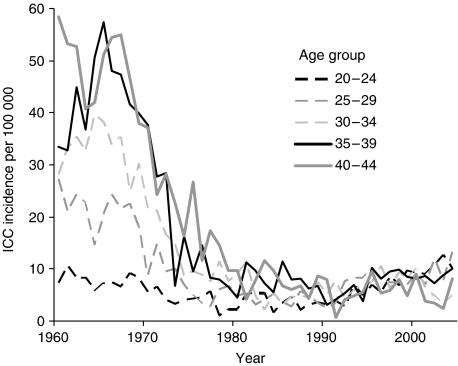
Cervical cancer incidence per 100 000 women per year in Finland by age at diagnosis. Data from the Finnish Cancer Registry.

**Table 1:** Percentage of each age that is sexually active in the model

Age	12	13	14	15	16	17	18	19	20+
Sexually active (%)	0.6	1.7	4.4	10.6	30.0	50.0	65.0	80.0	99–100

**Table 2:** Additional annual percentage of cervical cancer cases prevented by vaccinating male subjects in addition to female subjects by vaccination coverage and age

	**Age at vaccination**			
**Vaccination coverage (%)**	**12**	**15**	**18**	**21**
10	6.1	5.6	1.7	0.2
30	14.8	13.9	4.3	0.4
50	18.1	17.4	6.0	0.6
70	15.1	15.5	6.6	0.7
90	5.8	7.9	6.0	0.7

**Table 3:** Cumulative HPV16-associated cervical cancer cases prevented by year 2055 vaccinating female subjects only with 70% coverage with and without 3-year catch-up vaccination

**Age at vaccination**	**Cumulative cases prevented by 2055 no catch-up**	**Cumulative cases prevented by 2055 with 3-year catch-up**	**Additional cases prevented by 3-year catch-up**	**Percentage increase (%)**
12	1217.8	1382.4	164.6	13.5
15	1295.5	1408.3	112.8	8.7
18	776.2	830.9	54.7	7.1
21	385.9	422.8	36.9	9.6
